# Protocol for a randomised controlled trial evaluating the effectiveness of a CBT-based smartphone application for improving mental health outcomes in adolescents: the MobiliseMe study

**DOI:** 10.1186/s12888-022-04383-3

**Published:** 2022-11-30

**Authors:** S. H. Li, M. R. Achilles, M. Subotic-Kerry, A. Werner-Seidler, J. M. Newby, P. J. Batterham, H. Christensen, A. J. Mackinnon, B. O’Dea

**Affiliations:** 1 Black Dog Institute and School of Psychology, University of New South Wales, Sydney, New South Wales Australia; 2 Black Dog Institute, University of New South Wales, Sydney, New South Wales Australia; 3 Centre for Mental Health Research, Australian National University, Canberra, Australian Capital Territory Australia

**Keywords:** Depression, Anxiety, Cognitive behaviour therapy, Adolescent, eHealth, Mobile health, Mobile application

## Abstract

**Background:**

Depression is a leading cause of disability in adolescents, however few receive evidence-based treatment. Despite having the potential to overcome barriers to treatment uptake and adherence, there are very few CBT-based smartphone apps for adolescents. To address this gap, we developed ClearlyMe®, a self-guided CBT smartphone app for adolescent depression and anxiety. ClearlyMe® consists of 37 brief lessons containing core CBT elements, accessed either individually or as part of a ‘collection’. Here, we describe the protocol for a randomised controlled trial aiming to evaluate the effect of ClearlyMe® on depressive symptoms and secondary outcomes, including engagement, anxiety and wellbeing, when delivered with and without guided support compared to an attention matched control.

**Methods:**

We aim to recruit 489 adolescents aged 12-17 years with mild to moderately-severe depressive symptoms. Participants will be screened for inclusion, complete the baseline assessment and are then randomly allocated to receive ClearlyMe® (self-directed use), ClearlyMe® with guided SMS support (guided use) or digital psychoeducation (attention-matched control). Depressive symptoms and secondary outcomes will be assessed at 6-weeks (primary endpoint) and 4-months post-baseline (secondary endpoint). Engagement, conceptualised as uptake, adherence and completion, will also be assessed 6-weeks post-baseline. Mixed-effects linear modelling will be used to conduct intention-to-treat analyses to determine whether reductions in depressive symptoms and secondary outcomes are greater for conditions receiving ClearlyMe® relative to control at 6-weeks and 4-months post-baseline and greater for intervention adherers relative to non-adherers. To minimise risk, participants will be encouraged to use the Get Help section of the app and can also opt to receive a call from the team clinical psychologist at baseline, and at the 6-week and 4-month post-baseline assessments when reporting suicidal ideation.

**Discussion:**

This is the first clinical trial examining a CBT smartphone app specifically designed for adolescent depression. It will provide empirical evidence on the effects of ClearlyMe® on depressive symptoms when used with and without guided support.

**Trial registration:**

Australian New Zealand Clinical Trials Registry (ACTRN12622000131752).

**Universal trial number:**

U1111-1271-8519.

## Background

Depression is a leading cause of disability in adolescents and can compromise social functioning, academic performance and emotional development [1, 2]. As up to 25% of adolescents worldwide, at any given time, experience elevated symptoms of depression [3], timely access to effective interventions is critical to prevent chronic, lifelong illness [4]. Cognitive behavioural therapy (CBT) is a structured, skills-based psychotherapy typically delivered by a trained clinician over several sessions [5, 6]. It is the gold-standard psychological treatment for depression [7–10] and is recommended as a first-line treatment in children and adolescents aged 5-18 years [11]. However, many adolescents do not seek professional help due to a range of barriers [12–15]. Among those who do seek treatment, many do not receive CBT [16, 17]. As a result, treatment outcomes for depressed adolescents remain poor. The limited access and uptake of CBT is a major barrier that needs to be rectified.

Digital CBT interventions have been developed to overcome clinician shortages and other barries impeding access to treatment. These interventions been shown to be effective for lowering symptoms of depression and anxiety symptoms among adolescents [18–20]. Several recent meta-analyses examining the effectiveness of computerised, web-based, and smartphone app-CBT interventions in adolescents have shown there are small to medium effects in favour of digital interventions over no-intervention controls (e.g., wait list controls) in reducing depressive symptoms at post-treatment, however, these interventions did not appear to provide superior effects to therapeutically active comparators (e.g., group or individual CBT) [18–21]. Individuals’ engagement with digital interventions is also suboptimal, with low levels of program completion compromising users’ exposure to critical elements of treatment, such as cognitive restructuring [20, 22]. For example, reported completion rates in trials of digital CBT average 57%, with a range of 0 to 100%, demonstrating substantial variability between interventions [18]. If improved engagement and completion of digital CBT interventions increases exposure and development of critical CBT skills, as demonstrated in in-person CBT [23, 24], it may increase the benefits of digital delivery, however this requires empirical validation.

Low engagement with digital CBT among adolescents does not appear to be due to their unwillingness to use digital technologies to address mental health concerns. Uptake of commercially developed mental health smartphone apps is substantial, with millions of downloads and thousands of active monthly users [25]. Despite widespread uptake, many commercially developed apps lack both proof of effectiveness and critical CBT elements [26]. Among evaluated mental health smartphone apps, two recent reviews collectively identified only three smartphone apps for adolescents that contained elements of CBT, with none specifically targeting adolescent depression [27, 28]. Young people have reported that many of the existing computerised CBT interventions are less visually appealing than commercially developed apps, with unrefined usability and content that is not aligned with their needs [20]. This may explain why comparisons of studies show smartphone mental health apps outperform computerised and web-based interventions in terms of engagement, with an average retention rate of 80% [28] compared to 57% [18], although there is substantial variability in completion rates between apps. Given high rates of ownership and uninhibited access to internet and multimedia functionality, smartphones offer a promising means of delivering CBT that is yet to be adequately exploited.

While engagement with existing evidence-based digital mental health interventions is poor, human support (also referred to as guidance) has been associated with increased adherence to digital mental health interventions [29] and improved outcomes [18, 20, 21], however also see [19, 30]. Human support can take various forms. It may be provided by therapists or non-therapists either in-person, via telephone, chat sessions or email [18, 21]. Support may consist of encouragement to use the intervention, motivational interviewing, feedback on activity completion or therapeutic support to implement strategies [18, 21]. There is some evidence to suggest young people prefer text-based support over telephone calls [31, 32]. However, the positive impact of this specific form of support on outcomes, adolescent engagement in digital CBT, or its effect on factors associated with increased likelihood of using a mental health app, such as perceived need for help, and beliefs about app effectiveness and usability [33], is unclear [19, 32, 34]. Specifically, little is understood about the superiority of smartphone CBT with text-based guided support in terms of benefit or in promoting engagement and adherence relative to unsupported use [35]. Developing this understanding through robust research may inform how digital CBT could be offered to adolescents to optimise engagement [27, 28].

To overcome the gaps in current treatment availability, the Black Dog Institute developed ClearlyMe® - a free, self-directed CBT smartphone app that provides therapeutic content and symptom management strategies for mild to moderately severe symptoms of depression and anxiety in adolescents. Adolescents helped develop the app through extensive co-design, described in detail in Li et al. [36]. The app contains 37 brief (up to 10 minutes in duration), non-sequential ‘lessons’ consisting of psychoeducation, cognitive restructuring (thought challenging and behavioural experiments), emotion awareness and acceptance, activity scheduling, behavioural activation, goal setting, problem solving, exposure, relaxation, mindfulness, and values identification. The lessons encourage participants to practise the psychological skills between periods of app use and return to the app to reflect or complete further content as needed. Users are encouraged to complete the lessons via nine curated ‘collections’, which are a structured program of lessons grouped together to target a specific set of symptoms. Each collection varies in length, taking approximately 20 minutes to complete. Users can also complete the individual lessons from a ‘show all’ list whereby they self-select lessons considered to be relevant to their individual needs. In this list, lessons are categorised into three groups depending on their target: ‘emotions’, ‘thoughts’, ‘behaviours’. The app also includes a MoodCheck (i.e., rating mood on a scale from awful to great to track changes in mood over time), MindHacks (i.e., quick strategies that help in the moment), and Stories (i.e., short videos of young peoples’ experience managing mental health symptoms and positive help-seeking experience) to provide users with additional pathways to access therapeutic content. For these features, specific lessons are recommended after the feature is accessed. ClearlyMe® also includes in-app reminders, ‘saving’ and ‘favourite’ functions to support users to return to the app to reengage in content. The app also includes a ‘Get Help’ section that contains information of when and where to access additional mental health support services. ClearlyMe® is the first self-guided smartphone app providing a comprehensive CBT intervention developed specifically for adolescent depression. A rigorous clinical treatment trial among adolescents with mild to moderately-severe depressive symptoms is now needed to determine whether ClearlyMe® is effective for improving depressive symptoms and for engaging young people in depression treatment delivered via their smartphone.

### Trial objectives

This protocol is reported following SPIRIT guidelines to facilitate the reporting of the trial results using CONSORT guidelines [37]. It describes the methodology for the MobiliseMe study. The primary aim of the MobiliseMe study is to evaluate the effectiveness of the ClearlyMe® app for reducing depressive symptoms in adolescents. Using a three-arm parallel-group randomised controlled trial (RCT), the MobiliseMe study will compare self-directed use of the ClearlyMe® app with guided use alongside an attention-matched control condition to determine the effectiveness of the app for improving self-reported depressive symptoms in adolescents with mild to moderately-severe depressive symptoms after 6-weeks of use (primary endpoint) and at 4-month follow-up (secondary endpoint). This study will also evaluate the secondary impacts of the ClearlyMe® app on anxiety symptoms, psychological distress, emotional wellbeing, quality of life, rumination, emotion regulation and CBT skill acquisition. Participants’ engagement with the ClearlyMe® app (i.e., app uptake, treatment adherence and completions) and its influence on outcomes will also be explored. Creating and reporting multiple measures of engagement facilitates an understanding of the multiple ways participants may engage with the intervention, allows comparison between interventions and follows recent recommendations [38].

### Hypotheses

The primary hypothesis is that relative to the attention control condition, participants who receive the ClearlyMe® app (self-directed or guided) will have greater reductions in self-reported depressive symptoms between baseline and 6-weeks post-baseline (primary endpoint), as well as baseline and 4-month follow-up (secondary endpoint). A secondary hypothesis is that relative to the control condition, participants who receive the ClearlyMe® app (self-directed or guided) will report greater improvements in anxiety symptoms, psychological distress, emotional wellbeing, quality of life, rumination, emotion regulation and CBT skill acquisition between baseline and 6-weeks post-baseline, as well as baseline and 4-month follow-up. It is hypothesised that participants who receive guided support to use ClearlyMe® will report significantly higher levels of engagement than those who do not, however, no hypotheses have been made regarding the effect of guided support on outcomes given mixed findings in the literature e.g. [19, 21, 30, 35]. Exploratory analyses will examine whether the intervention was more effective for certain subgroups of the sample (e.g., based on age, symptom severity at baseline, greater perceived need for care, and higher openness to digital mental health) and whether the effects of the intervention were driven by other variables, such as rumination, emotion regulation, CBT skill acquisition, digital therapeutic alliance, and measures of app engagement.

## Methods/design

### Trial design

This superiority trial will utilise a three-arm, parallel group randomised controlled trial design with an equal allocation ratio. Outcome measures will be assessed at baseline, post-intervention (primary endpoint, measured at 6-weeks post-baseline) and follow-up (secondary endpoint, measured at 4-months post-baseline). A 4-month follow-up was selected to ensure participants in the control condition were provided with the intervention after a reasonable duration and to reduce participant attrition. The University of New South Wales is the sponsor of this clinical trial and ethics approval was given by the University of New South Wales Human Research Ethics Committee (HC#210889). This trial was registered with the Australian New Zealand Clinical Trials Registry on the 27th of January 2022 (ACTRN12622000131752) and has been allocated the Universal Trial Number U1111-1271-8519.

### Setting

This trial will be conducted entirely online, using the bespoke Black Dog Institute Research Engine, purpose built from which to conduct research trials. Data will be collected from individuals residing in Australia.

### Participants

Eligible adolescents are those aged 12 to 17 years old; located in Australia; who own or have access to a smartphone; have access to the Internet, an active email address and mobile phone number; comfortable with reading English at Grade 7-8 level; and who can obtain their parent or guardian’s consent. Eligible adolescents must also report mild to moderately-severe depressive symptoms as determined by a total score ranging between 5 and 19 on the Patient Health Questionnaire for Adolescents (PHQ-A) at screening. Ineligible adolescents are those who are currently receiving or about to start psychological treatment from a mental health professional for symptoms of depression or anxiety; are currently taking or about to start a course of prescribed medication for symptoms of depression or anxiety; report severe suicidal ideation as determined by a score of ≥2 on item 9 of the PHQ-A; respond ‘yes’ to items asking whether within the past month they have had serious thoughts or intentions about ending their life, or have made an attempt to end their life; nil or severe depressive symptoms as determined by a total score of ≤4 or ≥ 20 on the PHQ-A; or fail to satisfy any of the inclusion criteria at screening. Uptake of other depression interventions during the trial is permitted and will be assessed at primary and secondary endpoints.

### Interventions

Participants allocated to the intervention conditions (i.e., self-directed use of ClearlyMe® and guided use of ClearlyMe®) will be instructed to complete the ClearlyMe® app content by undertaking at least one collection per week (approximately 20 minutes in duration) for 6 weeks; however, participants can complete the app content in any order and according to their preferences. Participants will also be instructed to use the ‘Get Help’ section of the ClearlyMe® app if they feel they require extra mental health support during the intervention period, although this is not monitored by the research team. To promote compliance, participants allocated to this condition will be sent weekly SMS reminders (total of 6) to use the app. The ClearlyMe® app also includes an in-built ‘revisit the app’ reminder, which notifies participants to use the app after 7 days of inactivity. Participants who allow notifications during the app’s onboarding will receive these reminders but can deactivate it at any time via their mobile phone settings. Participants who fail to download the ClearlyMe® app within 7 days of randomisation will be sent one email and one SMS reminder to download the app. Participants who do not download the app will remain in the trial and be included in the intention to treat analyses.

Participants in the Guided use of ClearlyMe® condition will also receive a weekly guided support session delivered outside of school hours via Short Message Service (SMS) by a member of the research team using a standard script and decision flow chart (see Additional File 1). Research Assistants (RAs) will be trained to conduct these sessions and will be supervised by an experienced clinical psychologist. These sessions will consist primarily of technical support with some low intensity motivational coaching to encourage app use. The chat sessions are designed to take place in one sitting (i.e., approximately 20 minutes) but given the nature of SMS communication, will occur until the script is completed. Duration and script compliance will be recorded. Chat sessions will be prescheduled to take place between 3:30 pm and 6 pm on Mondays to Fridays. For non-responsive participants, the research team will instigate one additional contact attempt, made within 2 days of the initial attempt, however, the weekly chat schedule will remain unchanged, regardless of when a participant responds. Participants in this condition will be provided an additional $25 reimbursement for phone credit upon allocation to the guided support condition.

### Control condition

This study will utilise an active attention-matched control condition consisting of digital psychoeducation flyers. Attention-matched control conditions account for nonspecific intervention effects and are frequently used in other studies within the digital health field [39]. Psychoeducation is a known and acceptable intervention for young people that has demonstrated small positive effects on mental health without containing active CBT elements [40–42]. It is also information frequently consumed by young people during autonomous digital help seeking [36]. Digital psychoeducation in the current study has been designed to be comparable to the intervention conditions in terms of weekly dosage of content, activities, visual appeal, and duration to complete. These factors ensure digital psychoeducation satisfies both ethical and methodological requirements and has the potential to reduce control condition dropout [43]. Participants allocated to this condition will receive six weekly psychoeducation flyers, delivered via SMS that contains a URL to a static Portable Document Format (PDF). Participants are required to click the URL to access the flyer directly on their mobile device. The psychoeducation consists of six topics (taking approximately 20 minutes to read) containing information on mental health problems, links to credible Australian mental health organisations, and suggested activities for self-care. The content was created by the Black Dog Institute and used in other mental health studies among adolescents [41, 42]. Participants in this condition will not receive any additional contact from the research team, unless technical support (requested via email) is required. Engagement with the psychoeducation flyers will be determined by recording the number of times an individual participant clicks the flyer link, including time and date of access. Post-trial, participants in this condition will receive one email that includes a collated PDF containing all the psycho-education material in one file for future use. Participants will be instructed to use the ‘Get Help’ section of the study site if they feel they require extra mental health support during the study period, although this will not be monitored by the research team. All participants in the control condition will be provided with 6-weeks access to ClearlyMe® once their participation in the trial has concluded.

### Procedure and participant timeline

**Fig. 1:**
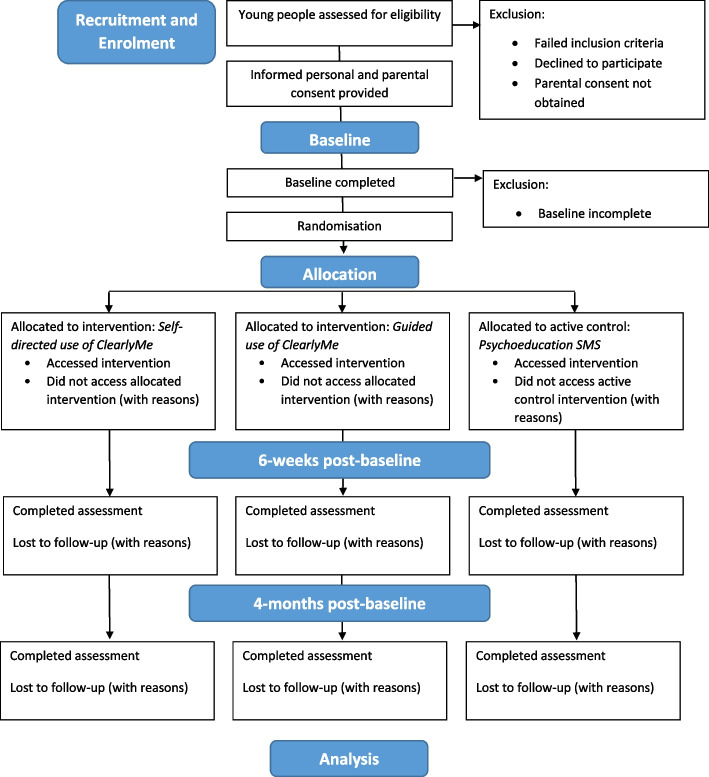
Study flow

Table 1 outlines the schedule of enrolment, interventions and assessments and Fig. 1 the study flow. Interested adolescents will be directed to the study webpage and asked to undergo a short online screener to determine their eligibility. Participants who do not pass screening will be provided with mental health service information. Only eligible participants will be invited to review the online Participant Information and Consent Form (PICF; see here) and proceed to the baseline assessment. Once completed, participants will be asked to register to the study by creating an account on the Black Dog Institute Research Engine. Participants are required to provide their full name, email, mobile phone number, date of birth, and to create a password. Registered participants will then be invited to view the online Parent Participant Information and Consent Form (P-PICF), to be completed by their parent or guardian within 1 week. Participants will receive three reminders to submit the P-PICF. Upon completion of the parental consent form, parents will be automatically emailed a copy of the P-PICF. If an invalid parent email address is entered, the research team will be notified via the study inbox and will contact the participant via email to confirm their parent’s details. Once the P-PICF has been submitted, participants will be instructed to complete the baseline assessment within 7 days. Participants who do not provide full consent or complete their baseline assessment within the allocated time will be automatically withdrawn from the study.

**Table 1:**
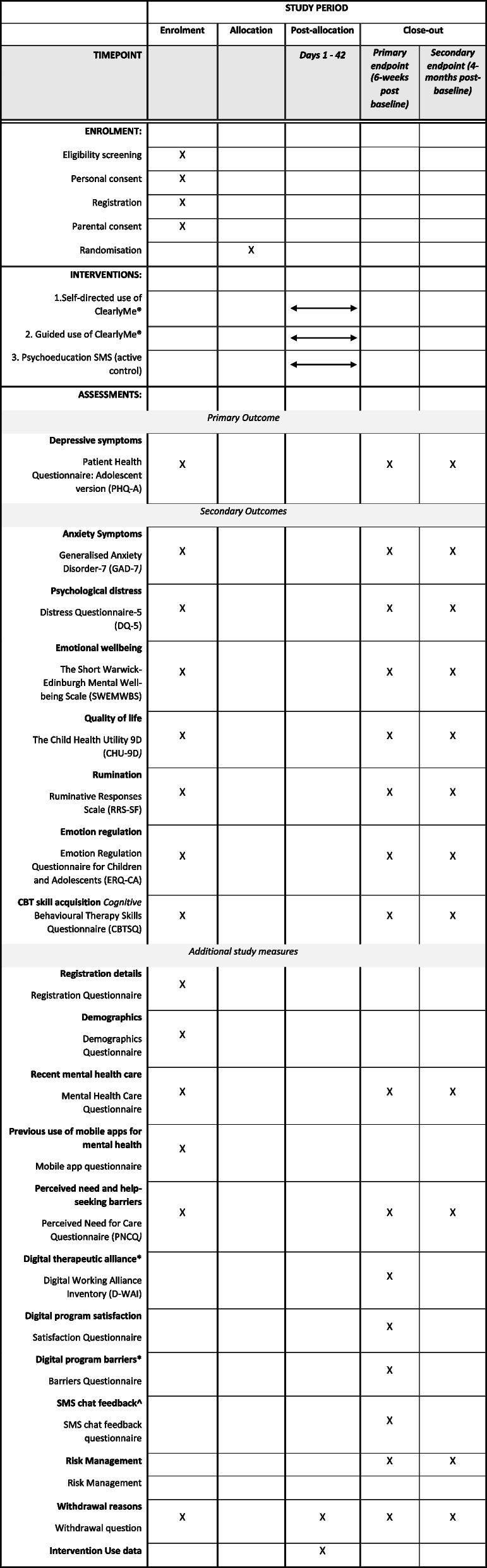
Schedule of enrolment, interventions, and assessments

^*^Assessed in intervention groups’ 1 and 2 only

^^^Assessed in intervention group 2 only

Upon completion of the baseline assessment, participants will be randomised to one of the three study conditions and receive the relevant intervention instructions via email and SMS. At 6-weeks post-baseline and at 4-months follow-up, all randomised participants will be invited via email and SMS to complete the study assessments. Participants will have 7 days to complete each assessment and will receive two reminders to do so. All study assessments will take approximately 20 minutes to complete and can be undertaken on any Internet-enabled device. Participants will be reimbursed 10AUD (electronic gift voucher) for each study assessment completed (30AUD total). In addition, participants receiving guided use of ClearlyMe® will receive 25AUD upon group allocation to cover the cost of sending and receiving SMSs. Discontinuation of the allocated intervention will occur if the participant withdraws from the trial or in the event that they are no longer able to engage in the intervention due to changed circumstances.

### Outcomes

#### Depressive symptoms

The primary outcome of this study is self-reported depressive symptoms as measured by the Patient Health Questionnaire-9 Adolescent version (PHQ-A) [44]. This 9-item self-report measure assesses the severity and frequency of depressive symptoms in the previous 14 days, with items rated on a 4-point Likert scale ranging from not at all (0) to nearly every day (3). Items are summed to produce a total score (range: 0 to 27) with higher scores indicating more severe depressive symptoms. Total scores will be used as the outcome measure, and can be classified into the following symptom severity categories: nil to minimal (0-4), mild (5-9), moderate (10-14), moderately severe (15-19), and severe (20-27). The PHQ-A is an adapted version of the validated PHQ-9 questionnaire [44, 45], has good psychometric properties for assessing depressive symptomology in adolescent populations [46–49] and has been endorsed for research and clinical evaluation with adolescents [44, 50, 51].

#### Generalized anxiety symptoms

This will be measured by the Generalized Anxiety Disorder-7 scale (GAD-7) [52]. This 7-item self-report measure assesses the severity and frequency of anxiety symptoms in the previous 14 days, with items rated on a 4-point Likert scale ranging from not at all (0) to nearly every day (3). Items are summed to produce a total score (range: 0 to 21) with higher scores indicating more severe anxiety symptoms. Total scores will be used as the outcome measure, and can be classified into the following symptom severity categories: nil to minimal (0-4), mild (5-9), moderate (10-14), moderately severe (15-19), and severe (20-27). The GAD-7 has good psychometric properties for assessing anxiety symptoms in adolescent populations [53, 54].

#### Psychological distress

This will be measured by the Distress Questionnaire-5 (DQ-5) [55]. This 5-item self-report measure assesses the frequency of psychological distress in the previous 30 days, with items are rated on a 5-point Likert scale, ranging from never (1) to always (5). Items are summed to produce a total score (range: 5 to 25) with higher scores indicating more severe psychological distress. The scale has established psychometric properties [55, 56] and has been used in adolescent populations [41, 57–60].

#### Emotional well-being

This will be measured by the Short Warwick-Edinburgh Mental Wellbeing Scale (SWEMWBS) [61, 62]. This 7-item self-report measures assesses individuals’ wellbeing in the previous 2 weeks, with items rated on a 5-point Likert scale, ranging from none of the time (1) to all of the time (5). Items are summed to produce a total score (range: 7 to 35) with higher scores indicating greater well-being. It has adequate psychometric properties to measure emotional wellbeing [63] in adolescent populations [64–66].

#### Quality of life

This will be measured by the Child Health Utility 9D (CHU-9D) [67]. This 9-item self-report measure assesses adolescents’ health related quality of life in nine domains of functioning including: worry, sadness, pain, tiredness, annoyance, schoolwork/homework, sleep, daily routine and ability to join activities. Each domain is rated on a 5-point scale, with each level representing increasing levels of severity within each domain e.g., I don’t feel worried today (1) to I feel very worried today (5). Items are summed to produce a total score (range: 9 to 45). The scale has good psychometric properties among adolescents [68–70] and can estimate quality adjusted life years for use in future cost-effectiveness evaluations of ClearlyMe® [71].

#### Rumination

This will be measured by the Ruminative Responses Scale – short version (RRS) [72]. This 10-item self-report measure assesses individuals’ tendency to engage in rumination. Individuals are asked to rate each item in response to the prefacing statement “what you generally do, not what you think you should do when feel down, sad or depressed”. The scale is composed of two subscales; reflection and brooding, with 5-items relating to each factor. Each item is rated on a 4-point scale ranging from almost never (1) to almost always (4). Items are summed to produce a total score (range: 10 to 40). Higher scores reflect higher levels of ruminative responses. The measure has good psychometric properties in adolescent populations [73–75].

#### Emotion regulation

This will be measured by the Emotion Regulation Questionnaire for Children and Adolescents (ERQ-CA) [76]. This a 10-item measure assessing individuals’ propensity to use cognitive reappraisal (6-items) and expressive suppression (4-items) as emotion regulation strategies. Items are rated on a 5-point scale, ranging from Strongly disagree (1) to Strongly agree (5). Items are summed on each subscale to produce total scores; cognitive reappraisal ranging from 6 to 30 and expressive suppression ranging from 4 to 20. Higher scores on each subscale reflect greater use of the corresponding emotion regulation skill. The measure has shown good psychometric properties for the assessment of emotion regulation strategies in children and adolescents [76].

#### CBT skill acquisition

This will be measured by the Cognitive Behavioural Therapy Skills Questionnaire (CBTSQ) [77]. The CBTSQ is a 16-item scale assesses the frequency of individuals’ use of cognitive behavioural skills. The measure is composed of two subscales measuring cognitive restructuring skills (9-items) and behavioural activation strategies (7-items). Items are rated on a 5-point scale, ranging from I don’t do this (1) to I always do this (5). Items are summed on each subscale to produce a total score for cognitive restructuring (range: 9 to 45) and for behavioural activation (range: 7 to 35). Higher scores on the subscales indicate greater use of CBT skills. The CBTSQ has good psychometric properties [77] and has been used in adolescent populations [78].

#### Demographics

Participants will be asked their gender identity (female, male, non-binary, different identity, free response option), whether they identify as Aboriginal or Torres Strait Islander (yes, no, prefer not to say), whether they identify as Lesbian, Gay, Bisexual, Transgender, Queer and/or Intersex (yes, no, I’d prefer not to say), their Australian state or territory location (New South Wales [NSW], Queensland [QLD], Victoria [VIC], Tasmania [TAS], South Australia [SA], Western Australia [WA], Northern Territory [NT], Australian Capital Territory [ACT]), location description (metropolitan, regional, or rural/remote), their date of birth, school grade (Years 7, 8, 9, 10, 11, 12, not currently in school) and the type of mobile device they use (iOS [e.g., Apple], Android [e.g., Samsung], other). Participants will also be asked how they heard about the study (e.g., social media, BDI website, word of mouth, other) and their motivations for participating (e.g., need for mental health care, desire to contribute to a broader social good, parents/carers encouragement, friend participating, financially motivated, desire to help research, interest in learning about mental health or other).

#### Recent mental health care

At baseline participants will be asked to report whether they have ever been diagnosed with anxiety or depression by a health professional (yes - depression only, yes - anxiety only, yes -both depression and anxiety, no, I don’t know, I’d rather not say), whether they have ever received psychological treatment for a mental health problem or mental illness from a health professional (yes, no, I’d rather not say) and whether they have ever taken prescribed medication for a mental health problem or mental illness like depression or anxiety (yes, no, I’d rather not say). At the primary and secondary endpoints participants will be asked to report whether throughout the study they were diagnosed with depression or anxiety by a health professional (yes - depression only, yes - anxiety only, yes -both depression and anxiety, no, I don’t know, I’d rather not say), whether they received psychological treatment from a health professional (yes, no, I’d rather not say), starting taking prescribed medication for a mental health problem or mental illness (yes, no, I’d rather not say) or felt like they needed help for a mental health issue like depression or anxiety (yes, no, I’d rather not say).

#### Previous use of mobile apps for mental health

Participants will be asked to report whether they have ever used any mobile app to help with their emotional wellbeing or mental health (yes, no, I’d rather not say), and if yes, whether they found it helpful (yes, no). Participants will also be asked how much they think their emotional wellbeing could be improved by using a mental health smartphone app on a 5-point scale ranging from not at all (1) to extremely (5).

#### Perceived need and help-seeking barriers

This will be measured by the Perceived Need for Care Questionnaire (PNCQ). The PNCQ is a four-item measure designed to determine an individuals’ perceived need for mental health care. Perceived need is assessed across four domains of adolescent mental health care: counselling, medication, information, and skill training. Each domain is assigned one of four levels of perceived need (need fully met, need partially met, need unmet, or no need) using the information provided regarding whether sufficient care was received. If perceived need is not fully met, participants select the type of help needed (e.g., counselling, medication, information, and skill training) and the barriers (e.g., self-reliance, stigma, accessibility, financial reasons) kept them from receiving adequate care. Barriers are categorised as attitudinal or structural (e.g., “Couldn’t get an appointment when needed [structural]” and “Wanted to work out the problem on my own [attitudinal; self-reliance]”) [15]. The instrument has good psychometric properties [79] and has been adapted for use in large representative samples of adolescents [80, 81].

#### Digital therapeutic Alliance (digital working Alliance inventory)

This will be measured by the Digital Working Alliance Inventory (DWAI) [82]. The DWAI is a 6-item measure adapted from the Working Alliance Inventory- short version to assess the therapeutic alliance between an individual and digital intervention (WAI-SF) [83, 84]. The measure assesses three core domains: goals (e.g., I trusted the app to guide me towards my personal goals), tasks (e.g., I believed the app tasks will help me to address my problem) and bond (e.g., The app encouraged me to accomplish tasks and make progress). Each item is rated on a 7-point scale ranging from strongly disagree (1) to strongly agree (7). Items are summed to produce a total score (range: 6 to 42). Higher scores reflect higher levels of digital working alliance with the program. The original scale has good psychometric properties [83, 84], and preliminary evidence of the DWAI’s psychometric properties is positive, however it is yet to undergo rigorous psychometric evaluation [85].

#### Digital program satisfaction

This will be assessed using an 11-item measure adapted from previous digital mental health research [59]. The measure is designed to assess participants’ satisfaction, experience and perceived helpfulness of the intervention received. For the first eight items on the scale, participants are asked to agree or disagree with a set of statements that assess aspects of their satisfaction and experience with the intervention. The measure also includes a helpfulness item, where participants are asked to rate the overall helpfulness of the intervention on a 5-point scale ranging from extremely unhelpful (1) to extremely helpful (5). The final two free response questions examine how ClearlyMe® or the psychoeducation flyers were helpful (e.g., In what ways did ClearlyMe help you?) and provide participants with the opportunity to suggest improvements (e.g., What would make ClearlyMe better?).

#### SMS chat feedback

The SMS Chat Feedback questionnaire is an 8-item measure that was adapted from [86] by the study authors to gain feedback on the SMS chat support provided to participants in the guided support condition. The first item on the scale asks participants whether they participated in the SMS chat support (yes, no). Participants who respond ‘no’ are asked what stopped them participating (free response). Participants who respond ‘yes’ are asked seven additional questions. Five are rated on a 5-point scale and assess how much participants liked the SMS chats (disliked a lot [1] to liked a lot [5]), perceived helpfulness for encouraging use of ClearlyMe® (extremely unhelpful [1] to extremely helpful [5]), convenience of the timing (extremely inconvenient [1] to extremely convenient [5]), and their level of satisfaction with the frequency and length of the SMS chats (not at all satisfied [1] to extremely satisfied [5]). Participants will also be asked two free response questions about what they liked or disliked about the SMS chats and how the SMS chats could be improved.

#### Digital program barriers

The Digital Program Barriers questionnaire is a 14-item measure was adapted from previous digital mental-health research conducted by researchers of this study [59]. This measure is designed to examine any barriers or difficulties that participants experienced accessing or using the ClearlyMe® app or the psychoeducation flyers. Each item requires participants to select “yes” or “no” in response to a statement describing a barrier, including technical issues, accessibility and individual factors.

#### Engagement – ClearlyMe®

The current study utilises and will report multiple measures of engagement, consisting of ‘uptake’, ‘adherence’, and ‘completions’. In the current study, ‘uptake’ is defined as the proportion of participants (%) who take up the app, measured by app downloads. ‘Adherence’ is defined as the proportion (%) of participants who followed the instructions for app use. This will be measured in two ways: 1) A categorical variable (adherers vs. non-adherers) in which participants who complete at least one collection each week for the entire 6 weeks will be deemed ‘adherers’; 2) A numerical variable in which participants will earn 1 point for each week that they adhered to the instructions (i.e., completed at least one collection) resulting in a total adherence score (range: 0 to 6). ‘Completions’ is used to describe participants’ exposure to the app content, measured in three ways: 1) the number of lessons completed as a proportion of the total number of lesson, 2) the proportion (%) who completed more than half of the app content (i.e, 19 of 37 lessons), 3) the proportion (%) that completed all of the app content (i.e., all 37 lessons). Additional exploratory data will be collected to describe participants’ use of the ClearlyMe® app: collections completed (out of 9), the total number of times app features are accessed (e.g., Stories, View all, Get help now, Mood check, Saved), time spent in app (minutes), the collections and lessons liked/disliked, the collections and lessons saved, and individual responses to the lesson activities. Different measures of engagement have been employed to move beyond the pervasive conceptualisation of engagement as the ‘more use, the better’ in acknowledgement that there are multiple ways participants may engage with the intervention [38, 87, 88]. Recommendations have been followed [88] to include measures frequently used in the literature (e.g., proportion of lessons completed), intervention specific measures (e.g., frequency of feature access) and a measure of adherence defined as the extent to which actual use aligns with instructions for use. This allows a deeper analysis of the most beneficial way to use ClearlyMe® and supports comparisons with other studies given the heterogeneous reporting of engagement [36, 38, 88].

#### Engagement – control condition

For participants allocated to the control condition, the number of times each psychoeducation flyer is accessed via the URLs in the weekly SMSs will be recorded for each participant and include time and date of access.

### Sample size

The total sample size required for detecting change in the primary outcome at the primary endpoint was calculated to be 489. This was based α = 0.05, power = 0.8, small to medium within-group effect sizes for psychoeducation (d = 0.10) [40, 41], self-directed CBT (d = 0.35) [89], and guided CBT (d = 0.65) [18, 19] on depressive outcomes and a conservative attrition rate of 20% between baseline and primary endpoint. This sample size will also be sufficient to detect small between-group effect sizes between the intervention conditions and the attention control condition and between the self-guided and supported use intervention conditions.

### Recruitment

Trial recruitment began on the 11th of May 2022, subsequent to submission of the trial protocol for peer-review. The first trial participant was enrolled on the 12th of May 2022. This study will utilise an online recruitment strategy. Study advertisements will be published on the Black Dog Institute website and social media channels (Facebook, Twitter, and Instagram) and provided to schools and organisations to share with their communities. Paid advertisements will be placed on social media sites (Twitter, Facebook, Instagram, Snapchat and Google). Online recruitment is an effective avenue to recruit generalised mental health samples [59, 90]. The research team will also contact relevant Australian mental health organisations and services to request distribution of study advertisements on their organisation’s communication channels (e.g., website, social media, newsletters, mailing list, in-person clinics). Study advertisements will also be emailed to individuals who have consented to being contacted about research studies conducted by the Institute. All study adverts will direct interested participants to the study website, where they will review the study information, and may choose to undertake screening, and provide consent using the provided links. Direct contact between the participant and study team is not required during recruitment and study enrolment. To ensure participant safety during recruitment, excluded participants are provided with contact information for Australian mental health and crisis support services and encouraged to seek help and speak to a trusted adult. The same information is provided to included participants immediately following consent, again at the end of the baseline survey and in the study welcome email. They are also encouraged to contact the research team if they have questions or are experiencing distress.

### Randomisation and blinding

Randomisation will be carried out according to the International Council for Harmonisation guidelines [91]. Randomisation to one of the three trial arms will be conducted immediately after completion of the baseline assessment using a computerised randomisation procedure within the Black Dog Institute Research Engine. A stratified randomisation approach with a block size of 6 (1:1 ratio) will be used to ensure balance across the conditions in terms of age (12 to 14 years vs. 15 to 17 years), and symptom severity (mild [as determined by a total score of 9 or less on the PHQ-A] vs moderate to moderately-severe [as determined by a total score of 10 or more on the PHQ-A]). Allocation will be fully automatic, with no input from the research team. Participants will not be directly informed of their condition allocation; however, they may be able to deduce this based on differences in the study activities. For the primary outcomes analyses, the statistician will be blinded to participants’ allocations. The statistician will be unblinded when examining intervention completion rates due to differences in the total number of modules/lessons across each condition as well as when conducting exploratory moderator analyses. Key members of the research team not involved in data analysis, including research assistants providing guided support, will be unblinded to participants’ allocation as they will require access to the Black Dog Institute Research Engine to contact participants if they experience adverse events and to provide support to participants allocated to the guided use condition.

### Data collection, management and statistical methods

All participant data will be collected and stored on the Black Dog Institute Research Engine, hosted on secure servers commissioned by the University of New South Wales at GovDC Data Centres located in Sydney, Australia. The data is stored in a SQL Server 2016 database which is backed up daily. The Research Engine automatically generates a unique participant identification code, to enable the protection of participant confidentiality. For analyses, all study assessment data will be exported, via a Microsoft Excel file, from the Research Engine to SPSS Version 26. These files will be stored on UNSW OneDrive and deidentified by approved members of the research team. All identifiable information, including participant name, email address, mobile phone number, IP addresses, and free response data, and group allocation will be removed from the outcomes analysis file. This dataset will then be transferred to the trial statistician for analyses using a password protected OneDrive file. All identifiable study assessment data will only be accessible to the research team listed on the project, while aggregate deidentified data will be retained for future evidence synthesis.

Primary analyses will be conducted to determine the effect of the interventions on depressive symptoms at the primary and secondary endpoints. Analyses will be undertaken on an intention-to-treat basis, including all participants randomised, regardless of adherence or intervention received. The effectiveness of the trial interventions will be established by change on the PHQ-A between baseline and 6-weeks post-baseline (primary endpoint) and baseline and 4-month follow-up (secondary endpoint), based on the interaction between time and condition, using mixed-effects repeated measures linear modelling with an unadjusted *p* value of 0.05. The mixed-effect linear modelling will account for all available data, under the missing at random assumption. Effect sizes will be calculated based on differences in observed change scores between baseline and 6-weeks post-baseline, using standard deviations of the change scores pooled across conditions. Attrition analyses will be conducted to determine whether missing data is associated with any baseline demographics (age, gender), mental health status (symptom severity, past or current treatment) or other descriptive variables. Any baseline variables identified as substantially imbalanced between groups will be added to the models on an exploratory basis to confirm the robustness of the findings to this imbalance. Where distributional assumptions cannot be satisfied, other modelling may be used to confirm the robustness of the findings. Similar models will be used for secondary outcomes. To analyse the effects of adherence on outcomes, the mixed-effects repeated measures linear modelling will be repeated for each intervention condition separately using the categorical adherence measure as a between group factor (adherers v non-adherers v control). The categorial adherence measure has been selected for this analysis to determine whether instructions for use optimise benefit as intended or require adjustment. These results will be published in the primary outcomes paper. Exploratory analyses will examine evidence for moderation and mediation, that is, whether the intervention was more effective for certain subgroups of the sample (e.g., based on gender, baseline symptom severity, perceived need for help and openness to digital interventions) and whether the effects on outcomes were influenced by other variables, including rumination, emotion regulation, CBT skill acquisition, digital therapeutic alliance, and measures of app engagement. These results may be published in separate papers to appropriately describe the methods and analyses. A deidentified data set will be made available upon reasonable request at the discretion of the principal investigator.

### Monitoring

The current trial will utilise a Trial Management Group to supervise the overall conduct and safety of the trial in accordance with the National Health and Medical Research Council (NHMRC) guidelines on Data Safety Monitoring [92]. Details about this group are published on the Australian New Zealand Clinical Trials Registry (see here). As this trial includes symptomatic adolescents, each study assessment will include a list of mental health services and support resources suitable for adolescents. Participants who report an Adverse Event (AE), which includes severe suicide ideation (indicated by a score of ≥2 on item-9 of the PHQ-A) reported at baseline or the primary or secondary end-points and severe depressive symptoms (indicated by a total score ≥ 20 on the PHQ-9-A) reported at the secondary endpoint, will be offered a follow-up call from a Clinical Psychologist who will conduct a risk assessment over the phone. If at risk, the participant will be advised to contact an appropriate service. Parents will be contacted if the risk of harm is deemed imminent or the participant requests it. All adverse events and serious adverse events will be formally recorded and reported to the Trial Management Group in a monthly report and to the University of New South Wales Human Research Ethics Committee by the Clinical Trial Manager. No interim analyses on primary or secondary outcomes are planned, unless it is warranted by safety reports or upon the request of the Trial Management Group. The Trial Management Group may recommend pausing or terminating the trial if they have concerns for participant safety, based on (but not limited to) a higher than anticipated rate of one or more of the primary endpoints. At any time, the University of New South Wales can audit trial conduct and this process will be independent from the investigators and the sponsor.

### Dissemination

A summary of the research results will be published on the Black Dog Institute website and emailed to all participants and their parents upon completion of the data analysis. Trial outcomes will also be prepared for publication in relevant peer-reviewed journals by the research team and presented at academic conferences. In accordance with the National Health and Medical Research Council open access policy, the researchers will endeavour to publish all papers from this research in open access journals. In all reports, participants will not be individually identifiable. Numerical data will be presented at the aggregate level. Any qualitative data reported will use the non-identifiable code allocated to it.

## Discussion

This protocol outlines the MobiliseMe study, a clinical trial which aims to examine the effectiveness of a new CBT-based smartphone app (ClearlyMe®) for reducing depressive symptoms and other mental health outcomes, including anxiety and wellbeing, in adolescents aged 12-17 years. ClearlyMe® was designed to address current gaps in treatment provision for depressed adolescents by creating a smartphone app containing a full course of CBT with qualities, such as visual appeal, usability and relevance, equivalent to popular commercially developed apps. The provision of CBT via a means that is accessible, youth friendly and retains users is critical to ensure effective early intervention, reduce dysfunction and prevent potential chronic disability across the lifespan [22, 27, 28]. If found to be effective, ClearlyMe® has the potential to provide an accessible, low cost, and scalable treatment to improve depressive symptoms in young people. This study addresses several gaps in the literature. It is the first evaluation of a CBT-based smartphone app targeting depression, designed with, and specifically for adolescents. It is also the first trial to compare the outcomes between users receiving guided support and those who do not, and to explore the factors that promote engagement and effectiveness. Outcomes of this trial will advance knowledge of how to support young people experiencing mental health symptoms using digital technologies and inform innovations in the field.

### Strengths

The design of the MobiliseMe trial is robust. First, the inclusion of an active-attention matched control condition will allow an evaluation of the impact of ClearlyMe® over and above the support young people commonly receive when help-seeking independently, that is, information about mental symptoms and treatment options [93]. Second, a comparison condition that involves using ClearlyMe® with SMS guidance is well-matched to the needs of young people [31, 32] and will provide much needed knowledge on the influence of guidance on engagement and effectiveness. Third, the sample size (*N* = 489) of the trial will ensure adequate power to detect intervention effects. Finally, a thorough consideration of ‘engagement’ and transparent reporting of how constructs such as uptake, adherence and completion have been operationalised, measured and analysed addresses a well-documented limitation in the field [22, 67, 88, 94].

### Limitations

Due to its scale, this study will not conduct clinical interviews to yield a formal diagnosis of a mental disorder. However, measures with well documented psychometric properties that provide clinically relevant classifications of adolescent depressive symptomology and severity, which are frequently used in the literature for this purpose, have been selected [51]. Engagement with, and effectiveness of ClearlyMe®, will be examined in the controlled environment of a research trial providing little indication of how the app will be used in less controlled environments, such as in the community. Additional research examining the use of ClearlyMe® will be required to determine whether adolescents use the intervention differently in real-world settings [95]. The 4-month follow-up assessment will not allow for conclusions to be drawn about the long-term sustainability of the effects of ClearlyMe®. The incorporation of longer follow-up periods in future studies will be required. Instructions on how to use ClearlyMe® (i.e., at least 1 collection each week) were determined by predicting use that would produce optimal therapeutic benefit based on clinical experience. While this may not prove to be the optimal way to use ClearlyMe®, this trial will allow an examination of patterns of use associated with benefit. Finally, while the purpose of this first evaluation is to determine whether ClearlyMe® is effective and safe, subsequent evaluations could include an assessment of its cost effectiveness.

Notwithstanding these limitations, outcomes of this trial will provide valuable insights into the potential of a new CBT smartphone app to fill a gap in current treatment provision and factors driving and associated with therapeutic benefit.

## Data Availability

Data sharing is not applicable to this article as no datasets were generated or analysed during the current study. A detailed study design and analysis plan were preregistered, including access to material, see here.

## Abbreviations

ACT: Australian Capital Territory
AE: Adverse Event
BDI: The Black Dog Institute
CHU-9D: Child Health Utility 9D
CBT: Cognitive Behavioural Therapy
CBTSQ: Cognitive Behavioural Therapy Skills Questionnaire
DWAI: Digital Working Alliance Inventory
DQ-5: Distress Questiionaire-5
ERQ-CA: Emotion Regulation Questionnaire for Children and Adolescents
GAD-7: Generalized Anxiety Disorder-7 sc
NHMRC: National Health and Medical Research Council
NSW: New South Wales
NT: Northern Territory
PHQ-A: Patient Health Questionnaire for Adolescents
PNCQ: Perceived Need for Care Questionnaire
QLD: Queensland
RCT: Randomised Controlled Trial
RRS: Ruminative Responses Sca
SA: South Australia
SMS: Short Message Service
SWEMWBS: Short Warwick-Edinburgh Mental Wellbeing Scale
TAS: Tasmania
TMG: Trial Management Group
UNSW: University of New South Wales
VIC: Victoria
WA: Western Australia
